# Home range variation of two different-sized groups of golden snub-nosed monkeys (*Rhinopithecus roxellana*) in Shennongjia, China: implications for feeding competition

**DOI:** 10.24272/j.issn.2095-8137.2018.044

**Published:** 2018-06-28

**Authors:** Peng-Lai Fan, Yi-Ming Li, Craig B. Stanford, Fang Li, Ze-Tian Liu, Kai-Hua Yang, Xue-Cong Liu

**Affiliations:** 1 College of Life Sciences, University of Chinese Academy of Sciences, Beijing 100049, China; 2 Institute of Ecology, College of Life Sciences, Beijing Normal University, Beijing 100875, China; 3 Key Laboratory of Animal Ecology and Conservation Biology, Institute of Zoology, Chinese Academy of Sciences, Beijing 100101, China; 4 Departments of Biological Sciences and Anthropology, Jane Goodall Research Center, University of Southern California, Los Angeles, California 90089, USA; 5 Shennongjia National Park, Shennongjia Hubei 442421, China

**Keywords:** *Rhinopithecus roxellana*, Home range size, Group size, Feeding competition

## Abstract

Knowledge on the home range size of a species or population is important for understanding its behavioral and social ecology and improving the effectiveness of conservation strategies. We studied the home range size of two different-sized groups of golden snub-nosed monkeys (*Rhinopithecus roxellana*) in Shennongjia, China. The larger group (236 individuals) had a home range of 22.5 km^2^ from September 2007 to July 2008, whereas the smaller group (62 individuals) occupied a home range of 12.4 km^2^ from November 2008 to July 2009. Both groups exhibited considerable seasonal variation in their home range size, which was likely due to seasonal changes in food availability and distribution. The home range in any given season (winter, spring, summer, or winter+spring+summer) of the larger group was larger than that of the smaller group. As the two groups were studied in the same area, with the confounding effects of food availability thus minimized, the positive relationship between home range size and group size suggested that scramble feeding competition increased within the larger group.

## INTRODUCTION

A home range is defined as the area in which animal individuals or groups spend their normal activities over a certain period in search of food and caring for young (Burt, 1943). Knowledge on the home range size of a species or population is of great importance for understanding its behavioral and social ecology (Isbell, 1991; Snaith & Chapman, 2007; Zhou et al., 2007) and for improving the effectiveness of conservation strategies (Bryant et al., 2017; Huang et al., 2017). The home range size of non-human primates (hereafter, primates) is influenced by a range of ecological and behavioral factors, including food availability and distribution (Curtis & Zaramody, 1998; Zhang et al., 2014; Zhou et al., 2007), group size (Dias & Strier, 2003; Gillespie & Chapman, 2001; Li et al., 2010), sleeping site location (Zhou et al., 2011), water availability (Scholz & Kappeler, 2004), parasite avoidance (Freeland, 1980), topography (Fan & Jiang, 2008), and intergroup relationships (Benadi et al., 2008).

Spatiotemporal distribution of food resources can affect the home range size both between and within primate species (Clutton-Brock & Harvey, 1977; Zhang et al., 2014; Zhou et al., 2007). Because leaves are more abundant and evenly distributed than fruits, folivores, e.g., most colobines, generally occupy smaller home ranges than frugivores, e.g., *Pan troglodytes* and *Ateles* spp. (Clutton-Brock & Harvey, 1977; Stanford, 1991; Zhou et al., 2007). For species inhabiting seasonal environments, the home range size of a group usually exhibits seasonal variation due to seasonal changes in food availability and distribution (*Eulemur mongoz*: Curtis & Zaramody, 1998; *Trachypithecus francoisi*: Zhou et al., 2007; *Macaca leonina*: Albert et al., 2013; *Hoolock leuconedys*: Zhang et al., 2014).

The influence of group size on home range size has also been widely investigated, but with contradictory findings; home range size increases with group size in many species and populations (Dias & Strier, 2003; Fashing et al., 2007; Gillespie & Chapman, 2001; Kurihara & Hanya, 2015; Li et al., 2010; Teichroeb & Sicotte, 2009), but not in all (Struhsaker, 1975; Whitesides, 1989). Theoretically, when food resources are limited, the addition of feeding members should reduce the amount of food intake per capita and thus larger groups are expected to occupy larger home ranges to obtain adequate food for all group members (Chapman & Chapman, 2000a; Isbell, 1991; Janson & van Schaik, 1988). Researchers often infer intragroup scramble feeding competition (i.e., reduction of resources without direct conflicts) from a positive relationship between home range size and group size (Fashing et al., 2007; Isbell, 1991; Snaith & Chapman, 2007). It should be emphasized that the influence of group size on home range size is highly dependent on food availability; if food resources are abundant enough to compensate for having more mouths to feed, more feeding members in larger groups may not lead to increased competition and thus home range expansion (Chapman & Chapman, 2000b; Isbell, 1991; Snaith & Chapman, 2007). Researchers often control the confounding effects of food availability by studying different-sized groups foraging in the same habitat (Dias & Strier, 2003; Gogarten et al., 2014). Unlimited food availability may explain the absence of a relationship between home range size and group size in some species or populations, particularly those that tend to rely heavily on leaves, an abundant food source (reviewed in Isbell, 1991).

The golden snub-nosed monkey (*Rhinopithecus roxellana*) is an endangered colobine species endemic to China and includes three geographically isolated populations: Qinling, Sichuan-Gansu, and Shennongjia (Li et al., 2002, 2007). Its long-term survival is threatened by habitat loss and fragmentation, illegal poaching, and increasing human activities due to the rapid development of ecotourism (Guo et al., 2008; Li et al., 2002; Xiang et al., 2011; Zhang et al., 2016). *R. roxellana* diverges from most colobines in various ecological aspects. Unlike most colobines living in tropical or subtropical forests, it inhabits temperate forests in mountainous areas at high altitude (1 000–4 100 m), which exhibit strong seasonality with cold and snowy winters (Kirkpatrick & Grueter, 2010; Li et al., 2002). Forest phenology and food availability strongly affect its diet, with flowers, young leaves, mature leaves, fruits, seeds, and buds becoming available and subsequently the main dietary components (Guo et al., 2007; Li, 2006; Liu et al., 2013b). Furthermore, lichens, an uncommon food source for primates, constitute an important part of its diet throughout (or almost) the year. *R. roxellana* lives in extraordinarily large multi-level groups of up to several hundred individuals, with one-male multi-female units as the basic social and reproductive level (Qi et al., 2014), whereas most other colobines live in small groups containing 3–20 individuals (Bennett & Davies, 1994; Oates, 1994).

Previous studies of the Qinling population have shown that groups of *R. roxellana* have home ranges much larger than those of most colobines (rarely >1 km^2^) (Li et al., 2000; Tan et al., 2007). For example, the West Ridge group in Yuhuangmiao (90 individuals) occupied a home range of 22.5 km^2^ from April to October 1995 and December 1996 to September 1997 (Li et al., 2000), and the East Ridge group (112 individuals) exploited a home range of 18.3 km^2^ from November 2002 to November 2003 (Tan et al., 2007). Both groups exhibited considerable variation in their seasonal home range size (Li et al., 2000; Tan et al., 2007). The home range size of the Shennongjia population has never been systematically studied. Su et al. (1998) provided the only preliminary report that the monkeys were observed in an area covering 40 km^2^ from November to December 1991, March to April 1992, and October 1992 to the end of 1995, but the study subjects were from different groups.

In the present study, we investigated the home range size of two different-sized *R. roxellana* groups in Shennongjia. Our results are of particular significance for conservation as the Shennongjia population has a smaller distribution area, a smaller population size, and lower genetic diversity compared to the other two populations (Li et al., 2007; Liu et al., 2015; Luo et al., 2012). In addition, because the two groups foraged in the same area and thus the confounding effects of food availability were minimized, intergroup comparisons of home range size bear important implications for intragroup scramble feeding competition.

## MATERIALS AND METHODS

### Study site

**Figure 1:**
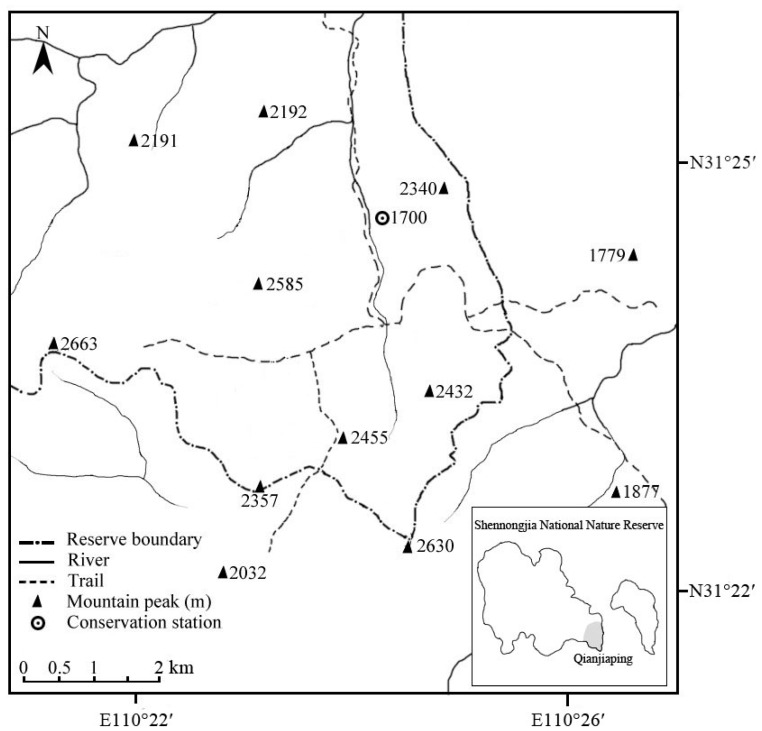
Topography of the Qianjiaping area of Shennongjia National Nature Reserve, China

Shennongjia National Nature Reserve (E110${}^{\circ }$03${}^{\prime }$–110${}^{\circ }$34${}^{\prime }$, N31${}^{\circ }$22${}^{\prime }$–31${}^{\circ }$37${}^{\prime }$) is separated into two parts by geographical features, main roads, and human residential districts (Figure 1). *R. roxellana* is found only within the western part and our study site, the Qianjiaping area (60 km^2^), is located at the southeastern end of this part. The topography of this area is extremely rugged, with an elevational range of 1 500–2 663 m (Liu et al., 2013b). The vegetation is characterized by deciduous broadleaf and evergreen conifer mixed forests. The climate is highly seasonal. The mean temperature at the elevation of 1 700 m is lowest in January (ca. –5.5 ${}^{\circ }$C) and highest in July (ca. 16.3 ${}^{\circ }$C). Annual rainfall is approximately 1 800 mm, with the rainy season occurring between July and September. Snow occurs from early November to middle March. Based on local climate, we defined spring from 21 March to 31 May, summer from 1 June to 31 August, autumn from 1 September to 31 October, and winter from 1 November to 20 March.

### Study groups

During the study period, the two different-sized groups of *R. roxellana* foraged mainly in the Qianjiaping area and occasionally in adjacent areas in the southeast (adjacent areas belong to Badong County). The larger group had been semi-habituated and studied periodically since 1999 (Li, 2001, 2002, 2004). We studied this group from September 2007 to July 2008, except for February 2008, and could approach it within 20–30 m. We lost this group at the end of July 2008 and did not contact it again in the study area during the study period. We looked for other potential monkey groups in the same area and found a much smaller one in November 2008. We followed this group through to July 2009, except for February 2009. Before this study, we had not observed this group foraging in the study area. The group had never been habituated or studied, and we could only approach it within about 100 m (the apparent differences in vigilance behavior indicated that this group was not a part separated from the larger group). Group sizes and compositions were determined when the monkeys crossed open areas or rivers, or when leaves of deciduous plants fell during winter (age-sex class definition following Li, 2007). During the study period, the larger group contained 236±38 individuals (*n*=8), including 106±12 adult males, 77±18 adult females, 35±10 juveniles, and 18±5 infants, whereas the smaller group contained 62±6 individuals (*n*=6), including 23±5 adult males, 22±3 adult females, 13±3 juveniles, and 4±3 infants. These counts may be biased because the monkey individuals were widely spread and our view was often obstructed.

### Data collection

While following each group, we estimated the central locations of group spread at half-hourly intervals and obtained GPS coordinates (longitudes and latitudes) via a portable GPS unit (Garmin GPSMap 60CSx 2.6-inch Mapping Handheld GPS). When we could not obtain a clear satellite signal due to dense canopy, we moved to a more open location (less than 20 m from the estimated group location). All study protocols adhered to the legal requirements of China and local management regulations of Shennongjia National Nature Reserve.

### Data analysis

We employed the fixed kernel density estimation (KDE) to calculate the home range sizes during various periods for each group via ArcView 3.3 with the Animal Movement Analysis Extension (Hooge & Eichenlaub, 1997). KDE has several advantages over grid systems and minimum convex polygons (MCPs) in estimating home range size (Caillaud et al., 2014; Fashing et al., 2007). KDE is less sensitive to sample size and the presence of outlying points. MCPs often overestimate home range size by including large areas never used, while grid systems largely depend on arbitrarily chosen grid dimensions. Therefore, KDE is also preferable for comparison analyses of home range size between groups or populations and has been widely employed to estimate home range size in primate studies (e.g., Caillaud et al., 2014; Fashing et al., 2007; Scholz & Kappeler, 2004). KDE computes the spatial utilization distribution based on a random sample of group locations (Seaman et al., 1999; Worton, 1989). We estimated home range sizes based on 95% volume contours of kernel probability density surfaces with a smoothing parameter selected by least squares cross validation, as used in other studies (Campera et al., 2014; Fashing et al., 2007; Hanya & Bernard, 2016; Scholz & Kappeler, 2004).

We first calculated the overall (i.e., annual for the larger group and winter to summer for the smaller group) and seasonal home range sizes for each group. For intergroup comparisons, we then estimated the home range size from winter to summer for the larger group. We also calculated the overlapping sizes of the above parameters (i.e., winter to summer, winter, spring, and summer home range sizes) between the two groups.

## RESULTS

**Figure 2:**
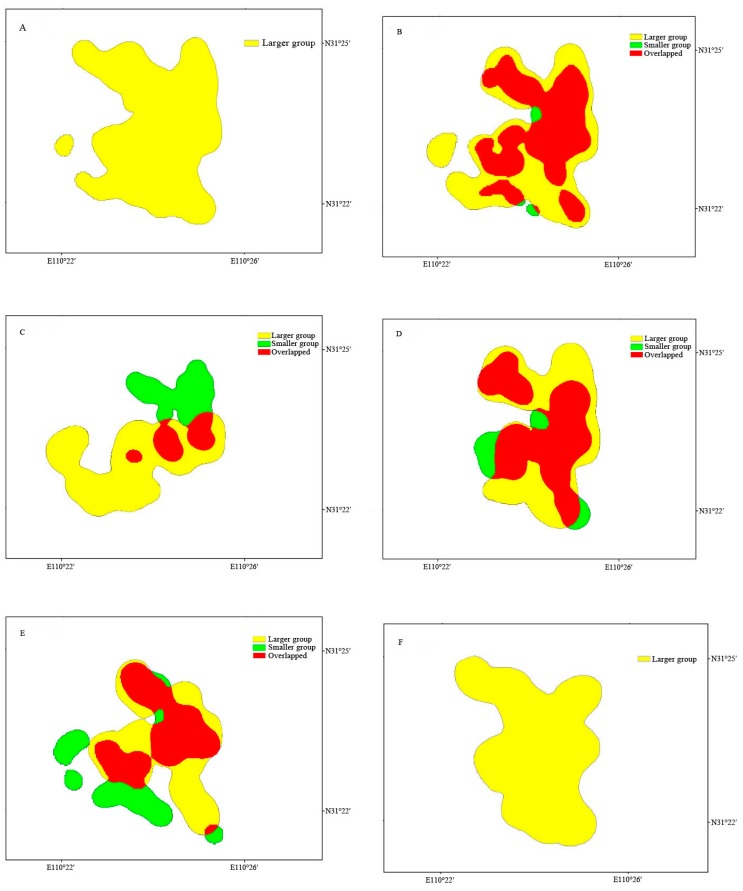
Home ranges of the two different-sized groups of *Rhinopithecus roxellana* in Shennongjia, China

We recorded 613 locations (winter: 192; spring: 137; summer: 106; autumn: 178) on 147 days for the larger group and 837 locations (winter: 258; spring: 303; summer: 276) on 130 days for the smaller group during the study period. The annual home range size of the larger group was 22.5 km^2^ (Table 1; Figure 2). The home range size of the larger group from winter to summer was 21.5 km^2^, much larger than that of the smaller group, 12.4 km^2^. Furthermore, the home range of the smaller group from winter to summer was almost entirely included within that of the larger group, with the overlapping range occupying 91.9% of the home range of the smaller group.

**Table 1:** Home range sizes (km^2^) of the two different-sized groups of *Rhinopithecus roxellana* in Shennongjia, China

	Period					
	Annual	Winter+Spring+Summer	Winter	Spring	Summer	Autumn
Larger group	22.5	21.5	12.3	18.6	14.5	19.4
Smaller group	N/A	12.4	6.0	12.0	11.7	N/A
Overlapped	N/A	11.4	2.2	10.7	7.9	N/A

Study period: September 2007 to July 2008 for the larger group, November 2008 to July 2009 for the smaller group. N/A: Not available.

Study period: September 2007 to July 2008 for the larger group, November 2008 to July 2009 for the smaller group. A: Annual; B: Winter+Spring+Summer; C: Winter; D: Spring; E: Summer; F: Autumn.

The seasonal home ranges varied in size in a similar pattern for both groups: autumn (for the larger group only) > spring > summer > winter (Table 1; Figure 2). On the other hand, for the larger group, spring and autumn home ranges were comparable in size and accounted for 82.7% and 86.2% of the annual home range, respectively. Winter and summer home ranges were much smaller and accounted for 54.7% and 64.4% of the annual home range, respectively. For the smaller group, spring and summer home ranges were similar in size and occupied 96.8% and 94.4% of the overall home range, respectively, whereas winter home range was much smaller and occupied 48.4% of the overall home range.

The home range of the larger group in any given season (winter, spring, or summer) was larger than that of the smaller group. Furthermore, spring and summer home ranges of the smaller group were largely included within those of the larger group, with the overlapping ranges accounting for 89.2% and 67.5% of the home ranges of the smaller group, respectively. In winter, the two groups foraged primarily in different areas, with the overlapping range occupying 17.9% of the home range for the larger group and 36.7% for the smaller group.

## DISCUSSION

Our study showed that the home range of *R. roxellana* in Shennongjia was large and comparable with that of the Qinling population (Li et al., 2000; Tan et al., 2007). It appears that colobines living in temperate habitats have larger home ranges than most other colobines in tropical or subtropical habitats, probably because food resources in temperate forests are less abundant and more spread out (Bishop, 1979; Grueter et al., 2008; reviewed in Kirkpatrick et al., 1998). For example, hanuman langurs (*Semnopithecus entellus*) in the Himalaya occupy home ranges up to 12 km^2^, whereas the home range sizes of the same species in Sri Lanka are only 2–3 hm^2^ (Bennett & Davies, 1994). The black-and-white snub-nosed monkey (*Rhinopithecus bieti*) is a colobine species endemic to temperate forests in China, and the home range size of a *R. bieti* group in the Samage Forest was reported to be 32 km^2^ over a 14.5-month period (Grueter et al., 2008).

Similar to the Qinling population, *R. roxellana* in Shennongjia exhibited considerable variation in home range size among seasons (Li et al., 2000; Tan et al., 2007). Seasonal changes in food availability and distribution were likely the primary driver for the seasonal variation in home range size in this study, as reported in the Qinling population (Li et al., 2000; Tan et al., 2007) and many other primates (Albert et al., 2013; Curtis & Zaramody, 1998; Zhang et al., 2014; Zhou et al., 2007). According to our previous studies in the same area, besides lichens, a fallback food available year-round, *R. roxellana* mainly eats young leaves and buds in spring, and fruits and pine seeds (*Pinus armandii*) in autumn (Li, 2006; Liu et al., 2013b, 2016). Our previous preliminary vegetation survey showed that these food types tend to be widely distributed across the study area (Yang et al., 2014), and thus the home ranges of *R. roxellana* in spring and autumn were larger than those in summer and winter. In summer, mature leaves are abundant and evenly distributed and become an important dietary component, and thus the home range largely contracted correspondingly. In winter, probably because of the shortage of food resources and/or being less active to save energy in the cold and snowy weather, the monkeys exploited the smallest home range.

The home range size during the period from winter to summer increased with group size. However, the two groups were studied in different years and thus the relationship between home range size and group size may have been confounded by food availability (Chapman & Chapman, 2000b; Isbell, 1991; Snaith & Chapman, 2007) as the availability of some important food types (e.g., pine seeds) can vary between years (Li, 2006). Therefore, it is more reasonable to infer the effects of group size from intergroup comparisons of seasonal home ranges. The home range sizes in winter, spring, and summer all increased with group size. In particular, spring and summer home ranges of the larger group were almost an expansion of those of the smaller group. In the study area, the abundances of important foods, including young leaves, mature leaves, buds, and lichens, in spring and summer (June to July in particular) are not likely to vary between years (Li, 2006; Liu et al., 2013b). Thus, we believe the confounding effects of food availability were not significant. These results suggested positive effects of group size on home range size, at least in spring and summer. The sensitivity of home range size to group size is supported by previous findings that food distribution density in the study area is quite low (Yang et al., 2014). Food plants occupying ≥5.0% of the seasonal diet account for only a small proportion of the total basal area (varying from 4.2% in summer to 11.5% in winter) and total shrub coverage (varying from 1.3% in autumn to 13.9% in spring). Furthermore, only 11.5% of trees and 18.9% of shrubs are encumbered by lichens.

The positive effects of group size on home range size suggested increased scramble feeding competition within the larger group. In addition to *R. roxellana*, *R. bieti* also has a large proportion of lichens in its diet (Ding & Zhao, 2004; Grueter et al., 2009a, 2009b; Huang ZP et al., 2017; Kirkpatrick, 1996; Xiang et al., 2007) and lives in extraordinarily large multilevel groups (Cui et al., 2007; Kirkpatrick, 1996; Kirkpatrick et al., 1998). It has been hypothesized that intragroup feeding competition is weak due to the ubiquitous availability, even distribution, and low quality of lichens (similar to mature leaves), thus allowing the formation of large groups (Grueter & van Schaik, 2010; Kirkpatrick, 1996). However, detailed data on the availability and distribution of both lichen and plant foods are still lacking for most populations of the two species. There is increasing evidence showing that intragroup feeding competition in these two species may be more significant than previously hypothesized; for example, several indices of foraging efforts increase with group size, including home range size (Li et al., 2010; this study), daily travel distance (Grueter & van Schaik, 2010), and time allocated for feeding and moving (Liu et al., 2013a).

Finally, it is worth mentioning that almost the whole home range of the smaller group was also used by the larger group during winter to summer in different years. This observation suggested that the quality of the range used by both groups might be higher than that of the surrounding range within the study area. Furthermore, there might be intergroup competition for this higher quality range and the smaller group filled the range only after the larger group ranged out of it. Intergroup feeding competition has seldom been investigated in *R. roxellana* probably because intergroup encounters are very rare due to the extremely large home ranges (Qi et al., 2014; Liu et al., unpublished data).
